# Chemotherapy of ovarian cancer in elderly patients

**DOI:** 10.7497/j.issn.2095-3941.2015.0077

**Published:** 2015-12

**Authors:** Tiffany A. Troso-Sandoval, Stuart M. Lichtman

**Affiliations:** Department of Medicine, Memorial Sloan Kettering Cancer Center, New York, NY 11725, USA

**Keywords:** Ovarian cancer, chemotherapy, paclitaxel, carboplatin, intraperitoneal, elderly, geriatrics

## Abstract

Epithelial ovarian cancer is primarily a disease of older women. Advanced age is risk factor for decreased survival. Optimal surgery and the safe and effective administration of chemotherapy are essential for prolonged progression-free and overall survival (OS). In this article, the available regimens in both the primary treatment and relapsed setting are reviewed.

## Introduction

Epithelial ovarian cancer is primarily a disease of older women. The median age at diagnosis is 63 years with 45.8% aged 65 years and older. In 2013, there were an estimated 222,400 new cases diagnosed and 14,030 will die of the disease^1^. Serous cancers which compromise 80%-85% of the tumors can manifest as ovarian, fallopian tube or primary peritoneal primaries^2^. Approximately 70% of patients will present at advanced stage and there is no effective screening test to detect disease at an early stage to improve survival. Risks to the development of epithelial ovarian cancer are associated with factors producing uninterrupted ovulation such as no prior pregnancy or low parity. Family history is a strong risk, especially those with a breast-ovarian cancer syndrome (BRCA-1 or -2) and hereditary non-polyposis colorectal cancer syndrome (HNPCC, Lynch II syndrome). In those situations, onset of disease had a mean age at diagnosis of 42.7 years but can occur at any age including over 70^3^^,^^4^. Age is associated with a marked decline in overall survival (OS). The reason for this is uncertain. Possibilities include diagnosis, undertreatment or biology^5^^-^^8^.

Older patients have problems which are often not present in the younger patient population. These including comorbidities, impairments in activities of daily living and instrumental activities of daily living, cognitive impairment and geriatric syndromes. One of the geriatric syndromes, polypharmacy, is particularly important when administering chemotherapy. There are issues of drug interactions, particularly with cytochrome P450 system, potentially leading to increase toxicity. Guidelines are being formulated for evaluation and minimize medication and improve outcomes^9^. The pharmacology of drugs used in ovarian cancer in older patients has been published as well as the effect of organ dysfunction^10^^-^^15^. Despite these issues most studies and subset analyses have shown older patients can tolerate chemotherapy at similar dose intensity as younger patients without a significant impact on quality of life (QOL)^16^. Physiologic age rather than chronologic age predicts toxicity associated with therapy. In particular, functional capacity, which does not correlate with Karnofsky Performance Score (KPS) or Eastern Cooperative Oncology Group (ECOG) performance status and comorbidity, is a useful predictor of the toxicity and benefit of systemic chemotherapy, as well as survival, morbidity and mortality in elderly cancer patients^17^. The older patients can derive maximal benefit from treatment with appropriate planning and supportive care. Interestingly, it has been postulated that clinical factors such as age, stage and tumor subtype have less of an impact on QOL than psychosocial factors such as family support and transportation. Further, several studies have sought to quantify and compare QOL in ovarian cancer patients^18^^,^^19^. A subset analysis of Gynecologic Oncology Group (GOG) 172 showed that personal well-being (PWB) when assessed on FACT-G was associated with a better overall outcome including an increased OS^20^.

This paper reviews the treatment of epithelial ovarian cancer with emphasis on older patients. While, much of the data is a subset analysis of larger studies in which older patients are underrepresented, prospective studies have been performed and are underway. There is no one definition of older or elderly. Studies originally used 65 years primarily due to Medicare data. Studies now focus on patients over the age of 70 years based on data showing these patients are at a greater risk of adverse events and vulnerability^21^.

## Primary treatment of ovarian cancer

### Chemotherapy post cytoreduction

With the aging of the population there will be an increase in the number of patients with epithelial ovarian cancer. A curative approach requires optimal cytoreduction followed by chemotherapy^22^. More than half of the newly diagnosed patients are over 65 years of age. Because of multiple factors including inadequate screening, nonspecific symptoms and tumor biology, most patients present at an advanced stage. Older patients have been shown to be less likely offered standard therapy, are more likely to develop toxicity and have poorer outcomes^4^^,^^23^^-^^26^. Unfortunately older patients are underrepresented in clinical trials and the presence of multimorbidity, polypharmacy and other geriatric factors make treatment challenging^27^. **Table 1** reviews the phase III trials of primary treatment.

**Table 1:** Review of phase III clinical trials for the initial therapy of epithelial ovarian cancer

Trial	Arms	No. of patients	Definitions of elderly	No. of elderly patients	Percentage of elderly patients (%)	PFS (months)	OS (months)
GOG 111^28^	CDDP-C *vs.* CDDP-P	386	NR	NR	NR	18 *vs.* 13	38.0 *vs.* 24.0
GOG 132^29^	CDDP *vs.* P *vs.* CDDP-P	614	60-69; ≥70	194, 108	32.0, 18.0	16.4 *vs.* 11.2	23 *vs.* 26 *vs.* 26.6
OVA-10 ^30^	CDDP-P *vs.* CDDP-C	680	NR	NR	NR	15.5 *vs.* 11.5	NR
Dutch/Danish Study^31^	CP *vs.* CDDP-P	208	NR	NR	NR	NS	NS
GOG 158^32^	CP *vs.* CDDP-P	792	61-70; 71-80; 81-90	215, 85, 10	27.0, 11.0, 1.0	20.7 *vs.* 19.4	57.4 *vs.* 48.7
AGO OVAR^33^^,^^34^	CP *vs.* CDDP-P	798	≥70	103	12.9	17.2 *vs.* 19.1	43.3 *vs.* 49.4
ICON2^35^	C *vs.* CAP	1,526	>65	482	32.0	15.5 *vs.* 17.0	33.0 *vs.* 33.0
ICON3^36^	CP *vs.* C/CAP	2,074	>65	591	29.0	17.3 *vs.* 16.1	36.1 *vs.* 35.4
JGOG^37^	Dose-dense CP *vs.* CP	631	>60	263	41.7	28.0 *vs.* 17.2	At 2 years: 83.6 *vs.* 77.7
GOG218^38^	CP + Bev + maintenance Bev *vs.* CP + Bev *vs.* CP	1,873	>70	430	23.0	14.1 *vs.* 11.2 *vs.* 10.3	NR
ICON7^39^	CP + Bev *vs.* CP	1,528	>70	150	10.0	19.0 *vs.* 17.3	NR
GOG 182^25^^,^^40^	CP ± gem/PLD/topo	3,686	≥70	620	16.8	15.4 *vs.* 16.4	39.6 *vs.* 44.2
Alberts *et al.*^41^	Cy + IP CDDP *vs.* CDDP-C	654	NR	NR	NR	NR	49 *vs.* 41
GOG114^42^	CDDP-P *vs.* C > P + IP CDDP	523	61-70; ≥70	114, 49	22.0, 9.0	23 *vs.* 28	52 *vs.* 63
GOG172^43^	CDDP-P *vs.* IP CDDP-P	415	61-70; >70	109, 48	26.0, 12.0	18.3 *vs.* 23.8	49.7 *vs.* 65.6

Note: PFS/OS was not reported in any paper specifically in older patients except GOG 182 with a PFS of 15 months and OS 37 months in older patients. Table modified from reference^44^. PFS, progression-free survival; OS, overall survival.

The combination of a taxane and platinum is a standard recommendation for primary chemotherapy in women following optimal or suboptimal surgical resection^22^. Current literature supports utilizing a combination of intravenous paclitaxel given every three weeks in combination with carboplatin. Alternatively a dose dense regimen of weekly paclitaxel with carboplatin is being used also in this setting^37^. The other standard in optimally cytoreduced patients is intraperitoneal chemotherapy^43^^,^^45^. This usually consists of intravenous and intraperitoneal paclitaxel and intraperitoneal cisplatin. Follow-up studies have demonstrated the continued efficacy of this approach^46^^,^^47^. However, this treatment regimen may be difficult for some older patients. Patient selection is critical to avoid excessive toxicity and allow patients to benefit from this treatment^48^. In cases of paclitaxel hypersensitivity, often due to the diluent Cremophor EL, docetaxel is an acceptable alternative unless the reaction is a class effect to taxanes^49^.

Since the year 2000, GOG study 158 has set the standard for primary chemotherapy. Results showed that carboplatin was better tolerated than cisplatin, yet provided comparable median free OS^50^. Despite that fact that this trial utilized an area-under-curve (AUC) of 7.5 (current recommendations AUC 5-6), the regimen was well tolerated with less than 10% non-hematological toxicity and an 87% completion rate. Of note, only 12% of the patients enrolled in this trial were over 70 years old and there was no subset analysis for this elderly population. In an attempt to improve on this doublet, a third drug was added in a randomized trial, GOG 182 (topotecan, liposomal doxorubicin, gemcitabine). The third drug did not add benefit but did add toxicity. There were no differences in outcomes in patients over 70 years compared to younger patients except for increased neuropathy and hematologic toxicity^25^^,^^40^.

Several trials have looked at utilizing weekly paclitaxel to increase PFS and OS with decreased toxicity compared to every 3-week paclitaxel. MITO-5 looked at a combination of weekly paclitaxel (60 mg/m^2^) and carboplatin AUC 2 on days 1, 8, 15, 21 of a 28-day cycle. An encouraging 88.5% of the elderly patients were treated without significant toxicity^51^. The JGOG 3016 trial from Japan was an important randomized phase III study looking at the same question of dose dense weekly paclitaxel and carboplatin *vs.* every 3-week administration. An improvement in both median PFS (28.2 *vs.* 17.5 months) and OS (100.5 *vs.* 62.2 months) was seen in the dose dense weekly group *vs.* the every 3-week group with a median follow up of 76.8 months^52^.

MITO 7 looked at every 3-week paclitaxel (175 mg/m^2^) and carboplatin AUC 6 *vs.* weekly paclitaxel (60 mg/m^2^) and weekly carboplatin (AUC 2) in a large multi-center-randomized trial and the regimens had comparable median PFS (17.3 *vs.* 18.3 months with the weekly schedule). Although the results of a superior median PFS with dose dense paclitaxel was not replicated in this trial compared to Japanese Gynecologic Oncology Group (JGOG), the weekly regimen did show less hematological and neurological toxicity. Smaller difference in median PFS in MITO 7 may be due to lower paclitaxel weekly dosing (60 *vs.* 80 mg/m^2^) in addition to weekly dosing of carboplatin (AUC 2 *vs.* q3 week AUC 6)^53^. The median age of patients was approximately 60 years. Patient age (above 70 years *vs.* below) did not affect efficacy. In suboptimally debulked patients, GOG 262 is evaluating every 3-week paclitaxel *vs.* dose-dense weekly paclitaxel in combination with carboplatin with or without bevacizumab.

The addition of bevacizumab to postoperative paclitaxel and carboplatin was explored in GOG 218 and ICON-7^38^^,^^39^. In GOG 218, patients were randomized to paclitaxel/carboplatin with or without bevacizumab and the third arm included bevacizumab maintenance. Although there was no significant difference between arms in OS, PFS was improved with the addition of bevacizumab 15 mg/kg given with paclitaxel and carboplatin followed by bevacizumab maintenance^38^. ICON-7 looked at the same combination in a 2 arm design (paclitaxel/carboplatin ± bevacizumab 7.5 mg/kg every 3 weeks with bevacizumab maintenance). This study included a greater population of optimally debulked patients (73%) and despite initially reporting an improvement of median PFS with the addition of bevacizumab, this difference shrank to a 1 month difference with data maturation. OS was also not increased overall in this trial. In spite of an exploratory analysis of patients with poor prognosis, there was a significant improvement in OS (34.5 *vs.* 39.3 months) with the addition of bevacizumab^39^.

Unfortunately, no subset analysis of older patients was performed in either GOG 218 or ICON-7 and therefore no direct conclusion can be drawn as to the safety and toxicity of this regimen in elderly patients. Other studies have demonstrated an increased risk of toxicity with bevacizumab in combination with chemotherapy in patients over 65 years old. In one study, patients who received bevacizumab were more likely to have grade 3-5 toxicity (78% *vs.* 57%), with the most common grade 3 toxicity being hypertension^54^^,^^55^.

### Elderly specific trials

GOG 273 was initiated in 2011 and is the first study to look at first line chemotherapy in elderly women and to assess both tolerance of chemotherapy and evaluate predictive characteristics that led to ability to complete treatment. Patients were evaluated with a geriatric assessment score to predict toxicity and for QOL. Treatment regimens were chosen by their physician (carboplatin AUC 5 *vs.* paclitaxel 135 mg/m^2^ and carboplatin AUC 5 *vs.* weekly paclitaxel 60 mg/m^2^ and carboplatin AUC 5). Arm 3 was added later in 2013 after first 2 arms had reached accrual. Preliminary data of the first 2 arms showed that patients chosen to be treated with every 3-week paclitaxel and carboplatin were younger and more fit, and had better rates of completion without dose delay or reductions. Patients chosen to receive single agent carboplatin were found to have lower rates of completion. Patients with limited social activities were also found to be less likely to complete chemotherapy. QOL was reported as improved in both arms of the trial^21^^,^^26^.

The treatment of elderly patients was addressed in the GINECO studies. A comprehensive geriatric assessment was used to stratify the patients’ ability to tolerate treatment. In EOC 1, cyclophosphamide and carboplatin were administered every 4 weeks with 6 cycles being completed in 72% of patients^56^. In EOC 2, paclitaxel (175 mg/m^2^) and carboplatin (AUC 5) were administered every 3 weeks^57^. The planned 6 cycles was completed in 68% of the patients and geriatric assessment was found to have prognostic value in predicting toxicity. Retrospective analysis of this data showed that use of paclitaxel in elderly patients did increase toxicity, specifically neurotoxicity. Older patients have been shown to have increased incidence of neurotoxicity^58^. Depression is another important factor found to have independent prognostic value. A geriatric vulnerability score was developed which can identify two groups with significantly different OS outcomes, treatment completion rates, grade 3–4 non-hematological toxic effects, serious adverse events and unplanned hospital admissions^59^. The clinical EWOC-1 uses the geriatric vulnerability score to define stage III/IV patients and treats them with carboplatin with or without paclitaxel (https://clinicaltrials.gov/ct2/show/NCT02001272; accessed December 14, 2015).

### Role of intraperitoneal therapy

In 2006, Armstrong *et al.*^43^ published their data from GOG 172 on intraperitoneal (IP) cisplatin and paclitaxel in stage 3, optimally cytoreduced ovarian cancer. This practice-changing regimen had significantly higher OS (68.6 *vs.* 49.7 months) when compared to intravenous (IV) paclitaxel and cisplatin. In this study, patients received a complex regimen of IV paclitaxel day 1, intraperitoneal cisplatin day 2 and IP paclitaxel day 8 in a 21-day cycle. The regimen has significant toxicity and only 42% of all patients were able to complete all 6 planned cycles. Of the 415 patients enrolled, only 12% were older than 70 years^43^.

In a retrospective case control study of elderly patients (>65 years old) who received IP therapy at Memorial Sloan Kettering Cancer Center (MSKCC) between 1994 and 2008, 54% of patients completed all 6 cycles and 75% completed at least 4 cycles. Only 12% required dose reductions. This study showed that IP chemotherapy could be given safely to older patients. Factors related choosing the appropriate older patients include evaluating performance status, functional status activities of daily living (ADLs), renal function, normal auditory function, and cardiac function^43^^,^^48^.

Recent studies have demonstrated the overall underuse of IV/IP therapy across all patients, but significantly less in patients over the age of 65^47^. Several factors are identified in this study as barriers to IP/IV administration of chemotherapy, one of which is age at time of diagnosis. In a prospective trial across multiple NCCN institutions, patients aged 55-64 years old (compared with patients of 18-54 years) had an odds ratio of receiving IP/IV therapy of 0.81^47^. In contradistinction, the OR for patients aged 65-74 years was 0.46 and only 0.11 for patients aged over 74 years old. Despite the established 16-month median OS data from GOG 172, there is a significant barrier to acceptance of IP/IV therapy as the standard of care in optimally debulked ovarian cancer patients, even under the age of 65. Placement of an IP port, risk of infection, institutional barriers to administration, inconvenience, and increased toxicity are all considered barriers. Additionally, some believe that dose dense paclitaxel with carboplatin, as in JGOG 3016, may potentially offer an alternative to IP therapy. Further studies will be needed to clarify this question.

### Maintenance therapies

Although primary cytotoxic chemotherapy has a high initial response rate, the median PFS is 16 months. Unfortunately, most relapsed cases are incurable and will ultimately result in death from disease^60^. Subsequent rounds of chemotherapy can cause accumulative side effects, which may also limit future quality of life and survival. Preventing recurrent disease or prolonging time to progression should therefore be a goal of future treatment approaches. Elder specific maintenance studies have not been performed.

The concept of maintenance therapy has been evaluated in several studies for patients that have had complete responses to upfront therapy. Paclitaxel was one of the first agents evaluated in this role to show an increase in PFS^61^. Updated results of maintenance paclitaxel monthly for 12 *vs.* 3 months showed a significant improvement for 12 monthly treatments (PFS 22 *vs.* 14 months and median OS 53 *vs.* 48 months)^62^. Neurotoxicity was a limiting factor with 23% of patients in the 12 monthly treatments suffering from grade 2 and 10% with grade 3/4. In the elderly population this risk of worsening neuropathic pain and motor dysfunction would most likely make this approach prohibitory.

Therapies targeted at VEGF have also shown some promise as maintenance^38^^,^^63^. In two phase III studies, GOG 218 and ICON 7, the continuation of bevacizumab after completion of standard 6 cycle of adjuvant chemotherapy with bevacizumab, showed a significant increase in median PFS. As discussed earlier, increased risk of hypertension was noted, but the risk of gastrointestinal (GI) bleeding was lower than previously reported.

In a non-cytotoxic, anti-VEGF approach, oral pazopanib was evaluated as a single agent in the maintenance setting in patients with advanced ovarian cancer that did not progress after initial chemotherapy^64^. This phase III trial randomized patients to pazopanib 800 mg daily *vs.* placebo and showed an increase of in 5.6-month PFS (17.9 *vs.* 12.3 months). No significant difference in OS was noted. Pazopanib was relatively well tolerated with most frequent toxicities including grade 1/2 hypertension, diarrhea, neutropenia or changes in liver function test (LFTs). Approximately 23% of the study participants were 65 years or older and patients up to age 85 were also included. Subset analysis based on age showed improved hazard ratio in the older subset although no breakdown based on age and toxicity was reported.

Future studies looking at PARP inhibitors, PIK3 inhibitors, AKT, mTOR, IGF-1R and other small molecule inhibitors are currently underway and many more agents are in development as the age of molecularly targeted oncology are incorporated in approaches for maintenance therapy^65^.

### Platinum sensitive relapse in elderly patients

Treatment options available at the time of relapse are based upon the extent of disease, the timing of the relapse with respect to initial therapy, and the patient’s performance status. Patients are considered to have platinum sensitive disease if the relapse occurs at 6 months or more following initial therapy. Surgical options at relapse are usually limited due to extent of disease and likelihood of further progression following surgery^26^^,^^44^. **Table 2** reviews the phase III trials of relapse. The paper of Teo *et al.*^44^ focuses on doublet therapy.

**Table 2:** Phase III trials of relapsed epithelial ovarian cancer

Trial	No. of patients	No. of elderly patients	Percentage of elderly patients	PFS (months)	OS (months)	PFS in elderly (months)	OS in elderly (months)
ICON4^66^	802	239	30	12.0 *vs.* 9.0	29.0 *vs.* 24.0	NR	NR
Intergroup^66^	356	100	28	8.6 *vs.* 5.8	18.0 *vs.* 17.3	Same as <65	NR
OCEANS^67^	484	178	37	12.4 *vs.* 8.4	NR	12.3 *vs.* 8.4	NR
CALYPSO^68^	976	157	16	11.3 *vs.* 9.4	NR	11.6 *vs.* 10.3	NR

PFS, progression-free survival; OS, overall survival.

Carboplatin can be used as a single agent in platinum sensitive relapse, particularly in patients with lower performance status. The risk of developing platinum refractory disease at a faster rate and concerns of lower response rates, led to an important study comparing monotherapy with a doublet containing paclitaxel. ICON 4 evaluated a combination of paclitaxel plus platinum based chemotherapy versus platinum alone. The combination therapy showed an increased median PFS (12 *vs.* 9 months) and OS (29 *vs.* 24 months). Neurological toxicities (grade 2-4) were higher with the combination, but interestingly the monotherapy had higher hematological toxicity. While almost one third of the cohort was over 65 years old, there was no age specific increase in toxicities in the older patients^66^. When repeated dosing of carboplatin is utilized, whether a single agent or combination, the issue of an acute hypersensitivity reaction must be considered. Various methodologies are being employed to try to reduce the incidence and modify the reaction^69^^,^^70^.

An EORTC intergroup trial evaluated the combination of gemcitabine and carboplatin *vs.* carboplatin alone in a cohort of 356 patients. Although this study was not powered to demonstrate an OS advantage, the combination therapy did show a significant increase in response rate (47.2% *vs.* 30.9%) and increase in PFS (8.6 *vs.* 5.8 months). The study included a QOL component that also demonstrated a better toxicity profile than paclitaxel and carboplatin doublet as well as no statistical difference in PFS related to age above or below 60 years^71^.

Further investigation of carboplatin-based doublets was evaluated in the CALYPSO trial comparing pegylated liposomal doxorubicin with paclitaxel and carboplatin in a cohort of 976 patients. This non inferiority trial showed that the liposomal doxorubicin arm had superior PFS (11.3 *vs.* 9.4 months) with no significant difference in hematological toxicities, but had less non-hematological toxicities such as hypersensitivity reactions and peripheral neuropathy. In a subset analysis of patients over 70 years (median age 74), the toxicity profiles remained in favor of non-taxane arm with less alopecia, neuropathy, arthralgias and febrile neutropenia. The pegylated liposomal doxorubicin arm had more hand foot syndrome compared with the paclitaxel arm, as this is a known side effect of the drug. Overall, carboplatin plus pegylated liposomal doxorubicin was found to have a better therapeutic index in patients over 70 years of age^72^.

The role of bevacizumab in the setting of platinum sensitive ovarian recurrence was investigated in the OCEANS trial. Gemcitabine and carboplatin was compared to be the same with addition of bevacizumab^67^. The combination with bevacizumab showed an increase in PFS (12 *vs.* 8.4 months) and an increase in response rate (78.5% *vs.* 57.4%). Older patients were well represented in the cohort with the median age 60-61 years old and more than 35% of patients over 65 years. Toxicities associated with the addition of bevacizumab included grade 3 hypertension (17.4% *vs.* 1%) but there was no specific breakdown in over 65-year-old patients. Notably, there were no bowel perforation or GI bleeding reported for patients while on treatment. Recently, a final update of OS and safety was reported which did not show any new safety concerns but also showed no significant increase in OS (33.6 *vs.* 32.9 months) after median follow up of over 58 months^73^.

### Platinum resistant relapse in eldly patients

Patients are considered to have platinum resistant disease if they suffer a recurrence within 6 months from primary chemotherapy. In this setting, patients are often retreated with a non-platinum single agent such as weekly paclitaxel, liposomal doxorubicin, gemcitabine, topotecan, pemetrexed or vinorelbine^26^. There are not any studies looking specifically at this situation in the elderly patient. Single agent paclitaxel has an expected response rate of 10%-25% with a median duration of response ranging 4 to 8 months. Gemcitabine, topotecan and liposomal doxorubicin are often preferred agents given their favorable toxicity profile in the elderly population. Treatment in this setting is strictly palliative, so toxicity considerations as well as maintenance of QOL are paramount.

Bevacizumab has been utilized as a single agent, non-chemotherapy option in the recurrent setting. In a phase II study, the GOG looked at the response rate and safety profile of bevacizumab in patients with recurrent or persistent disease. In a group of 62 patients with a median age of 57, there was a 21% clinical response rate (2 complete, 11 partial; median response duration, 10 months), and PFS greater than 6 months of 40.3%. Unfortunately there was no age related subset analysis performed. Primary toxicities included grade 3 hypertension (9.7%), grade 3 venous thromboembolism (1.6%), but no GI perforation^74^.

The use of bevacizumab in the elderly population has raised concerns of increased risk of thromboembolic events, hypertension and GI perforation^54^. Subsequently, the increased morbidity from hypertension and thromboembolism may also be increased in elderly overall, but particularly heightened in patients with multimorbidity.

Previously reported risk of bowel perforation may have been originally overestimated. In a single institutional retrospective analysis of 160 patients with recurrent ovarian cancer treated with bevacizumab, there was only a 4% incidence of bevacizumab associated GI perforation^75^. Additionally, in a prospective phase III trial of chemotherapy in advanced ovarian cancer, the addition of bevacizumab was shown to only increase risk of bowel perforation by 1.4% (2.8% *vs.* 1.2%)^38^. Nevertheless, neither of these studies contained risk related to age.

The addition of bevacizumab to a chemotherapy agent was evaluated in the platinum resistant recurrent setting in AURELIA, a large, randomized phase III open label trial. In this study, the investigator would choose the chemotherapy agent for the patient from pegylated liposomal doxorubicin, weekly paclitaxel or topotecan, and then patients were randomized to receive single agent chemotherapy versus addition of bevacizumab. The primary endpoint of PFS was slightly improved with the addition of bevacizumab (6.7 *vs.* 3.4 months) and overall response rate was also increased with combination (27.3% *vs.* 11.8%). There was no difference in OS in this poor prognosis cohort^76^.

## Conclusion

The treatment of high grade serous ovarian cancer in elderly patients requires careful assessment of the patient’s functionality. Despite the high percentage of patients over the age of 65 (>50%) who develop high grade serous ovarian cancer, very few studies have specifically analyzed efficacy and toxicity in the elderly patient, or have included only small percentages of patients from this age group.

Additionally, most studies did not include a geriatric assessment which encompasses not just a Karnofsky performance status but also assesses important factors such as number and severity of comorbidities, living conditions, cognitive evaluation, nutritional status and presence of other geriatric syndromes such as dementia, delirium, depression and falls. In selected elderly patients, chemotherapy can be administered both safely and effectively. There are several regimens available to consider for the elderly patient and careful attention should be given in selecting the appropriate combination therapy with respect to side effect profile. GOG 273 is actively evaluating the efficacy and toxicity of chemotherapy specifically in the elderly population. This important prospective study also includes a comprehensive geriatric assessment and we anxiously await the results of this trial.

Future research utilizing small molecule inhibitors and targeted therapies will need to include the elderly, over 65-year-old population, more comprehensively as well. Current technologies that enable molecular profiling of tumor tissue will become invaluable in selecting appropriate targeted therapy. PARP inhibitors, PIK3CA inhibitors and mTOR inhibitors as well as WEE-1 kinase inhibitors from the p53 pathway, are likely to become important therapies in the treatment of ovarian cancer. These inhibitors and many others are actively being studied in both the relapse setting as well as in upfront therapy. Many of these exciting new therapies may afford improved efficacy and less toxicity in the elderly population.
